# Supplementary material for: Health experiences of pregnant and women with chagas disease in the Atacama, Tarapaca, and Metropolitan regions of Chile. Mistreatment as an indicator of healthcare barriers
Source: PLoS One. 2024 Nov 8;19(11):e0313498. doi: 10.1371/journal.pone.0313498 (PMC11548719; doi:10.1371/journal.pone.0313498)
Supplement: Attachment — Submitted filename: Response to rewieurs.docx [file pone.0313498.s001.docx]

Response to Reviewers. September 10, 2024.

Dear Reviewers,

We sincerely appreciate the review, as well as your comments and suggestions. We hope that our revisions have enriched the text accordingly. We have incorporated most of your recommendations, which contribute to a better understanding of the document.

We have attached the revised article file with track changes enabled to highlight the modifications made.

Below, we respond to each of the reviewers' and editors' requests. We have copied each recommendation and provided our response beneath it, indicated with an "R." (in red)

We would like to express our gratitude once again for the valuable comments and suggestions provided by the reviewers. Andrea and Camila

Editors:

1. Title changes requested: The title has been modified as per the request.
2. Regarding informed consent: It has been clarified that informed consent was obtained in written form pre-pandemic, and verbally during and after the pandemic, with the record of this information preserved in the audio of the interviews.
3. The email of the Ethics Committee has been provided as the contact point to receive any requests for the data if needed.
4. The title of the online form has been changed.

Revisor #1:

1. Line 12. Replace “forms of Chagas transmission worldwide” with “forms of Trypanosoma cruzi transmission worldwide”. Clarify throughout the text that it is the parasite, not the disease itself, that is transmitted.

R: Line 12 has been revised and changed to “forms *Trypanosoma cruzi* transmission worldwide.”

2. Throughout the manuscript: Correctly refer to "Chagas disease" instead of just "Chagas", the name of the discoverer.

R: I have corrected the term “Chagas” to “Chagas disease” throughout the text. However, I have used the term “Chagas problem” when discussing the disease from a broader perspective related to the implications of carrying Trypanosoma cruzi without having developed Chagas disease.

3. Line 16. Italianize “Trypanosoma cruzi” consistently throughout the manuscript.

R: *Trypanosoma cruzi* has been italicized throughout the text.

4. Line 39. Clarify whether Chile is free of vector transmission caused by domiciled species only (e.g., Triatoma infestans) or all Triatominae species. Consider discussing potential vectorial transmission by Mepraia spp.

R: The issue regarding the significant Triatoma species in Chile, relevant to the transmission of *T. cruzi*, has been clarified in the text.

According to the objective of the national vector control program, the focus has been on controlling the native domiciliary vector, *Triatoma infestans*, a species of epidemiological importance in Chile and the southern cone. In this context, the vector transmission of *T. cruzi* through *Triatoma infestans* has been interrupted. In the national context, there are no records of evidence of *T. cruzi* transmission through wild vectors.

5. Line 71. Ensure reference [20] is correctly placed at the end of the sentence.

R: The reference on Line 71 has been correctly placed at the end of the sentence.

6. Line 127. Include median age and interquartile range.

R. Given the characteristics of the study, I do not consider these data significant enough to incorporate. But the median is 32. And the Q1 : 28 and Q3 36 the RIC is 8.0 (36-28)

7. Line 128. Specify the other two South American countries with similar participant characteristics due to the small sample size.

R: This information has been incorporated.

8. Line 128 (and others). Spell out numbers below thirteen for consistency.

R: All numbers have been kept in numerical format.

9. Line 126-131. Consider presenting these results in a table format for clarity.

R. It is considered that presenting the data in text format is clearer than using a table, as the highlighted information relates to various topics, making it difficult to consolidate all the information in a single table.

10. Line 133. Use "Chagas" with capitalization throughout the manuscript, honoring Dr. Carlos Justiniano Ribeiro Chagas.

R. Capitalization has been applied throughout the text when referencing "Chagas.".

11. Line 151. Remove the unnecessary repetition of "as".

R. It has been removed.

• Line 268: Insert a space between "repeated" and "across".

R. A space has been added.

• Line 362. Clarify "standard 162" as it appears to be a typographical error.

R: It has been revised and corrected. Technical Standard No. 162 refers to the technical guidelines and normative issued by the Ministry of Health to address the problem of Chagas disease at the national level.

Reviewer #2:

1.Thank you for the work you have done in Chile to bring attention to migrant women who are living with Chagas disease who are either pregnant or postpartum. The manuscript is otherwise written well, and results are easy to interpret. I have some additional suggestions to help improve the manuscript as it stands and prior to publication:
- Please review for grammatical errors. Please be consistent when discussing "Chagas" and would recommend either leaving as "Chagas disease" throughout manuscript or abbreviate as "CD".

R: We have reviewed the manuscript for grammatical errors and corrected those detected.

We have changed "Chagas" to "Chagas disease" where appropriate. We used "Chagas issue" in a few instances to refer to it from a broader perspective. In the original manuscript, we used the concept of "Chagas" with the understanding that it represents a complex and multidimensional issue that affects both the individual and the community, extending beyond the mere development of the disease. Nevertheless, we have decided to take your suggestion into account and use "Chagas disease" throughout most of the manuscript, except in the parts mentioned above.

2. I would consider how you use "vertical transmission" and "vertical Chagas". Many people get confused between vertical and vectorial transmission as the terms are very similar appearing. "Congenital Chagas disease" is a better term to be used when discussing the disease itself. I recommend adding "congenital Chagas disease" to keywords

R. We have corrected the text to refer to “congenital Chagas disease” to avoid any potential confusion.

3. in the abstract on line 15-16, "carriers of Trypanosoma cruzi" is not a great way to describe someone living with Chagas disease. I would re-word this sentence and possibly utilize "living with Chagas disease"

R. We have rephrased that paragraph.

4. I suggest adding a figure that contains a map of the three regions where the study was conducted. Within the map, I would also suggest adding population density and catchment. What is the "Metropolitan Region"? More clarification on the sites is important. You can add the clinics to the map.

R: We have incorporated a map that shows the location of the three regions and includes the most significant data related to the diagnosis of pregnant women in those regions, as well as data on migrants in each region and at the national level.

5. please clarify recruitment of the patients in lines 99-102. Which healthcare institutions? I would add participating clinical institutions to the "acknowledgements".

R: We have rephrased the text as follows: The participants were selected by the treating healthcare teams at the hospital or primary care level in each region. These teams facilitated the first contact with the women. Following this, the local project teams contacted the women by phone, confirmed their participation, and obtained informed consent. All participants had to have been diagnosed positive for Chagas disease after 2016, when Technical Standard n°162 was implemented in the Chilean health system.

We would like to thank the Chilean Ministry of Health and the Seremi, health services, reference hospitals, and Cesfam centers of the Atacama, Tarapacá, and the Metropolitan regions.

6. Line 100: diagnosed with "Chagas disease"

R: Corrected

7. - Did the team assess if the participants had concerns for transmission in Chile, Bolivia or other regions of south America? Where did the other two migrant from in South America? Was there concern for congenital Chagas in the participants or other where they coming from regions of vectorial transmission?

R: The majority of the women interviewed were from Bolivia, Chile, Argentina and Venezuela. The interviewing team identified concerns related to information on congenital Chagas disease and barriers associated with health communication.

8. The title needs attention. After reading the manuscript it appears that many of the participants were "migrants" and I would suggest adding to the title somehow. There is a major focus throughout the study on migratory health and I think it should be added.

R: The title has been changed.

9. another question about the title is "pregnant" women. It appears that interviews occurred during pregnancy and postpartum periods. May consider adding "Pregnant and Postpartum Women..."

R: It has been changed in the title and in the text.

10. I would consider discussing stigma in those living with Chagas. <https://www.ncbi.nlm.nih.gov/pmc/articles/PMC3772024/>
<https://academic.oup.com/trstmh/article/114/7/476/5734980>

R: The text has been modified to include the contribution of qualitative social research in better understanding the social barriers, stigma, and exclusion related to Chagas disease, as well as the importance of addressing the social components that affect individuals living with this disease.
